# Global DNA methylation patterns in Alcohol Use Disorder

**DOI:** 10.1590/1678-4685-GMB-2023-0139

**Published:** 2024-01-08

**Authors:** Jaqueline B. Schuch, Cibele E. Bandeira, Jorge L. S. Junior, Diana Müller, Mariele F. Charão, Bruna S. da Silva, Eugenio H. Grevet, Felix H. P. Kessler, Lisia von Diemen, Diego L. Rovaris, Claiton H. D. Bau

**Affiliations:** 1 Universidade Federal do Rio Grande do Sul, Faculdade de Medicina, Departamento de Psiquiatria, Programa de Pós-Graduação em Psiquiatria e Ciências do Comportamento, Porto Alegre, RS, Brazil.; 2 Universidade Federal do Rio Grande do Sul, Hospital de Clínicas de Porto Alegre, Centro de Pesquisa em Álcool e Drogas, Porto Alegre, RS, Brazil.; 3 Universidade Federal do Rio Grande do Sul, Instituto de Biociências, Departamento de Genética, Programa de Pós-Graduação em Genética e Biologia Molecular, Porto Alegre, RS, Brazil.; 4 Universidade Federal do Rio Grande do Sul, Hospital de Clínicas de Porto Alegre, Programa de Psiquiatria do Desenvolvimento, Porto Alegre, RS, Brazil.; 5 Universidade Feevale, Programa de Pós-Graduação em Toxicologia e Análises Toxicológicas, Novo Hamburgo, RS, Brazil.; 6 Universidade de São Paulo, Instituto de Ciências Biomédicas, Departamento de Fisiologia e Biofísica, São Paulo, SP, Brazil.

**Keywords:** Substance use disorder, addiction, alcohol dependence, 5-methylcytosine

## Abstract

Alcohol Use Disorder (AUD) is a highly prevalent condition worldwide that produces a wide range of pathophysiological consequences, with a critical impact on health and social issues. Alcohol influences gene expression through epigenetic changes mainly through DNA methylation. In this sense, levels of 5-methylcytosine (5-mC), namely Global DNA methylation (GMe), which can be influenced by environmental and hormonal effects, represent a putative biological mechanism underlying alcohol effects. Our aim was to investigate the influence of AUD diagnosis and alcohol patterns (i.e., years of addiction, use in the last 30 days, and alcohol severity) on GMe levels. The sample consisted of 256 men diagnosed with AUD and 361 men without AUD. DNA samples from peripheral blood were used to assess GMe levels, measured through the levels of 5-mC using high-performance liquid chromatography. Results from multiple linear regression analysis indicated that the presence of AUD was associated with lower GMe levels (beta=-0.155, p=0.011). Other alcohol-related outcomes were not associated with DNA methylation. Our findings are consistent with the hypothesis that the impact of chronic and heavy alcohol use in GMe could be a potential mechanism mediating the multiple organ damages related to AUD.

## Introduction

Alcohol Use Disorder (AUD) is characterized by physical and behavioral symptoms that involve craving, relapse, and alcohol tolerance ( American Psychiatric Association, 2013), besides several impairments in social and health areas. The worldwide prevalence of current alcohol use, defined as alcohol consumption in the past 12 months, is 43% (more than two billion people) ( World Health Organization, 2018), with similar estimates observed in Brazil ( Bastos et al., 2017). Additionally, about 13% to 18% of Brazilian individuals have heavy alcohol drinking ( Garcia and Freitas, 2015; Bastos *et al.*, 2017; World Health Organization, 2018). In Brazil, a nationwide study also showed a prevalence of AUD ranging between 2 and 0.7%, being lower in the southern region, and higher among men ( Bastos *et al.*, 2017). In 2016, the harmful use of alcohol resulted in more than three million deaths per year, almost 5.3% of all deaths in the world ( World Health Organization, 2018). Therefore, a substantial global burden of diseases is attributed to frequent or heavy alcohol consumption.

The chronic use of alcohol has a broad range of pathophysiological consequences in the organism that cause an extremely high impact on the healthcare systems ( World Health Organization, 2018). There is growing evidence that alcohol can alter the epigenome, mainly through methylation processes ( Resendiz et al., 2014; Zhang and Gelernter, 2017). This dynamic process is reinforced by the evidence that alcohol withdrawal and recovery are also related to changes in DNA methylation ( Witt et al., 2020). Although differential methylation at specific sites is a commonly used approach, Global DNA methylation (GMe) has emerged as a tool to assess the overall impact of extensive biological pathways that interact with the genome and exposome. GMe has been associated with neuropsychiatric disorders ( Jiang et al., 2017; Müller et al., 2021, 2022), where even minor variations in GMe could be part of pathophysiological processes ( Breton et al., 2017). The gold standard method to estimate GMe levels is the evaluation of the overall 5-methylcytosine (5-mC) levels relative to total cytosines in the genome using the high-performance liquid chromatography (HPLC) technique ( Chen et al., 2013). In this sense, a slight disparity of 0.1% in GMe levels between two groups - cases and controls - could represent a significant difference of over 28,000 cytosines that are methylated distinctly throughout the human genome (which contains more than 28 million CpG sites) ( Luo et al., 2014). However, up to now, only AUD studies using GMe proxy-based approaches were performed, observing both hypermethylation ( Bönsch et al., 2004; Kim et al., 2016) or hypomethylation in individuals with AUD compared to controls ( Soundararajan et al., 2021).

Considering the growing evidence that GMe is involved in the pathophysiology of psychiatric disorders and may suffer effects from environmental hazards ( Barbosa et al., 2019; Müller et al., 2022), this biological marker is a logical candidate for AUD studies, once chronic alcohol use involves at the same time an environmental stressor (alcohol effect in cells and organs) and a mental disorder (AUD). Therefore, we aimed to assess the association between AUD and GMe levels through HPLC. We also evaluated the influence of alcohol use patterns (i.e., years of addiction, use in the last 30 days, and alcohol severity) on DNA methylation levels.

## Subjects and Methods

### Sample

This cross-sectional study included 256 men with AUD according to the Diagnostic and Statistical Manual of Mental Disorders, fifth edition (DSM-5) criteria (American Psychiatric Association, 2013), and 361 men without AUD, from the metropolitan region of Porto Alegre, Southern Brazil. Individuals with AUD consisted of 209 inpatients under a specialized clinic for detoxification treatment in a male addiction unit at the Hospital de Clínicas de Porto Alegre (HCPA) and 47 men from the Attention-Deficit Hyperactivity Disorder (ADHD) Outpatient Clinic at HCPA. Cases must fulfill the following inclusion criteria: aged 18 years or older, have a diagnosis of AUD according to DSM-5, and be men. Non-AUD individuals were selected from the Blood Blank (n=199) and the ADHD Outpatient Clinic at HCPA (n=162). All individuals considered non-AUD had either a negative screening for AUD and other substance use disorders through self-report or lack of diagnosis through clinical psychiatric assessment. Non-AUD individuals also had to be men, aged 18 years or older. Individuals with neurodegenerative diseases and/or severe neurological conditions that may affect the cognitive process were excluded from the study. Sample characteristics are presented in Table 1. 

**Table 1 -:** Sample characteristics.

	AUD (n=256)	non-AUD (n=361)	Statistics	p-value
	Median [IQR]			
Age (years)	49.0 [40-56]	29.0 [23-37]	U=14.89	<0.001
Schooling (years)	9.0 [6-12]	12.5 [11-15]	U=-11.81	<0.001
	Mean (SD)			
GMe levels (%)	3.42 (0.48)	3.53 (0.41)	t=2.775	0.006
	n (%)			
Skin color (white)	174 (68.0)	360 (99.7)	X^2^=127.01	<0.001
Marital status (married)	86 (34.0)	125 (34.6)	X^2^= 0.01	0.939
Nicotine use^a^	200 (78.7)	94 (26.0)	X^2^=163.86	<0.001
Major depressive disorder^a^	48 (24.9)	90 (24.9)	X^2^=0.01	0.999
Generalized anxiety disorder^a^	10 (7.0)	19 (9.5)	X^2^=0.73	0.392
Bipolar disorder^a^	23 (11.9)	21 (5.8)	X^2^=5.50	0.019

^a^Lifetime use or a lifetime disorder. AUD: alcohol use disorder, IQR: interquartile interval, SD: standard deviation. Data were expressed as median [IQR], mean (SD) or n (%).

This study is in accordance with the Declaration of Helsinki guidelines and was approved by the HCPA Ethics Committee (2014-0249, 2016-0600). All individuals signed an informed consent form.

Sociodemographic data were obtained through detailed questionnaires by trained and supervised interviewers. Psychiatric comorbidities were assessed using the Structured Clinical Interview for DSM Disorders ( First et al., 2002). Each individual in our sample underwent evaluation by psychiatrists and met diagnostic criteria for the described psychiatric disorders according to the DSM-5. The Addiction Severity Index version 6 ( Kessler et al., 2012) was applied to inpatients and provided data regarding alcohol use patterns. The highest score obtained from the Clinical Institute Withdrawal Assessment for alcohol revised (CIWA-Ar) was used to estimate the severity of withdrawal symptoms ( Laranjeira et al., 2000), and the number of standard drinks per day was calculated as previously defined (i.e., one standard drink is equal to 340 mL for beer, and 40 mL for distilled spirits) ( Carneiro *et al.*, 2016).

Individuals with AUD, admitted to the HCPA for detoxification treatment, had blood drawn for analysis of liver function. Blood collection was carried out in the morning while fasting within the first 24 hours of hospitalization. Hepatic function parameters, including gamma-glutamyl transferase (GGT), alanine aminotransferase (ALT), and aspartate aminotransferase (AST), were measured in the Biochemistry Department at the Clinical Analysis laboratory of the HCPA, following standard procedures.

### Quantification of global 5-mC levels (GMe)

DNA samples were obtained from peripheral blood (5 mL) using the salting-out technique ( Lahiri and Nurnberger 1991). The DNA was quantified, and quality parameters were checked using the NanoDrop™ spectrophotometer equipment. DNA with 2 μg (volume 200 μL) was used to measure GMe, following the protocol from Barbosa et al. (2019). Sample treatment was based on Ramsahoye (2002) and all laboratory procedures for calibration and reproducibility assessment were applied ( FDA, 2022). Briefly, the procedure consisted of the enzymatic removal of RNA, followed by DNA hydrolysis. The treated samples were injected into the HPLC-DAD equipment, and the nucleoside separation was conducted in a C-8 column (150X4.6 mm, 5 µm), monitored at 280 nm. The methylated cytosines (5-mC) were expressed as a percentage, considering the total deoxycytidine (dC) amount. The following equation was applied: GMe = [5mdC/(dC + 5mdC)] * 100.

### Statistical analyses

A multiple linear regression model was performed to assess the effect of AUD on GMe levels. Potential confounders were selected based on previous evidence of influences in methylation and/or showed significant differences between the groups evaluated. Therefore, the multiple linear regression model included the following variables: age, sample origin (blood bank, addiction unit at HCPA, or clinical sample of ADHD), schooling, skin color, nicotine use, and bipolar disorder. In individuals with AUD, the relationship between DNA methylation levels and alcohol-related outcomes (e.g., age of first use, years of use, severity, and use in the last 30 days) was analyzed through Spearman correlations.

Chi-square, t-test, or Mann-Whitney was used to compare sociodemographic data, nicotine use, and psychiatric comorbidities between subjects with and without AUD. All analyses were performed using SPSS software version v.18 (Chicago, IL, USA).

## Results

Sample characteristics are presented in Table 1. Individuals with AUD were older, non-white, with less education, and presented a higher prevalence of nicotine use and bipolar disorder. Among these individuals, GMe levels were not correlated to alcohol-related outcomes, including age of first use (r=0.053, p=0.524), use in the last 30 days (r=0.084, p=0.314), years of use (r=-0.004, p=0.961), and addiction severity (ASI-6 score, r=0.110, p=0.181, Table 2). The median number of daily standard drinks was 25, which was not correlated to GMe levels ( Table 2). Liver function parameters were also not correlated to GMe levels (p>0.05, Table 2).

**Figure 1 -:**
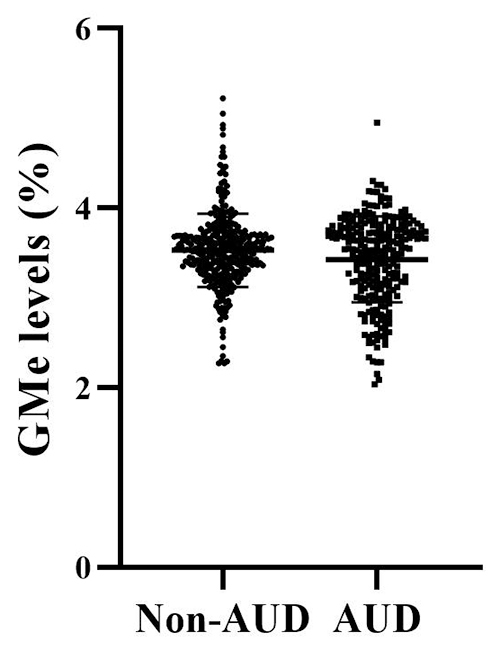
GMe levels in non-AUD and AUD individuals.

Multiple linear regression analysis showed an association between AUD and GMe levels (beta=-0.155, p=0.011, Table 3). Individuals with AUD presented lower GMe levels than non-AUD individuals ( Figure 1, Table 1). Skin color was also associated with GMe levels (beta=0.115, p=0.021). However, age, schooling, nicotine use, and the presence of bipolar disorder presented no effect on GMe levels in our sample.

**Table 2 -:** Alcohol-related outcomes and GMe levels.

		GMe levels	
	Median [IQR]	r Spearman	p-value
Age of first use (years)	15.0 [13-18]	0.053	0.524
Use in the last 30 days	29.5 [20-30]	0.084	0.314
Years of use	25.0 [14-33]	-0.004	0.961
Alcohol severity (ASI-06)	65 [60.5-70.5]	0.110	0.181
CIWA-Ar score	4.00 [1-6]	0.039	0.595
Standard drinks (daily)	25 [16.7-50]	0.021	0.765
Aspartate transaminase	37.0 [24.0-77.0]	0.021	0.768
Alanine transaminase	32.0 [20.0-56.7]	0.001	0.991
Gamma-glutamyl transferase	83.0 [39.7-160]	0.014	0.848

**Table 3 -:** Multiple linear regression model assessing the effect of AUD on GMe levels.

	Beta	t-Statistics	p-value
Alcohol Use Disorder	-0.155	-2.554	0.011
Sample origin^a^	0.014	0.274	0.784
Age	0.007	0.136	0.892
Schooling	-0.034	-0.652	0.514
Skin-color	0.115	2.309	0.021
Nicotine use	-0.028	-0.563	0.573
Bipolar disorder	0.063	1.454	0.147

All variables were included in a single multiple linear regression model. ^a^Blood bank, addiction unit at HCPA or clinical sample of ADHD.

## Discussion

In our study, we observed lower levels of GMe in individuals with AUD compared to subjects without AUD (p=0.011). This hypomethylation state was previously correlated to higher alcohol consumption and associated with AUD through epigenome-wide association studies (EWAS) and methylation proxy-based approaches ( Zhang et al., 2013; Friedel et al., 2020; Dugué et al., 2021; Soundararajan et al., 2021). Considering that alcohol consumption can alter methylation patterns that might affect gene expression, epigenetics could represent a biological mechanism by which alcohol impacts physiological functioning in the organism.

The fact that alcohol is a methyl donor inhibitor and reduces DNA methyltransferase (DNMT) ( Chen et al., 2013) and S-Adenosyl methionine (SAM) levels ( Mato and Lu, 2007; Hamid *et al.*, 2009), thus modifying and reducing methylation processes, provides a biochemical basis to support our findings. In addition, alcohol also induces oxidative stress processes, which inactivate methionine adenosyl-transferase and other DNMTs, leading to a reduction in methylation reactions ( Carretero et al., 2001). Individuals with AUD also have deficits in important nutrients, such as folate and other B-complex vitamins, that act as cofactors to methyl transfer reactions. In this sense, preclinical studies showed that deficient diets increased ethanol consumption ( Williams et al., 1949) and alcohol exposure induced changes in folate homeostasis ( Chen *et al.*, 2013), in a bidirectional process. Alcohol use can also generate DNA damage, which repair could lead to demethylation of 5-mC ( Chen *et al.*, 2011). Furthermore, chronic alcohol use has an impact on several organs, and it is an important risk factor for the development of alcoholic liver disease, which is characterized by steatosis and inflammation in the liver. This condition has also been associated with alterations in several epigenetic mechanisms, including global DNA hypomethylation ( Mandrekar, 2011). Notably, most of the cytokines throughout the genome are methylated, especially in heterochromatin regions ( Li and Zhang, 2014). This explains why hypomethylation can be so critical since it “releases” the genome, generating genomic instability. Furthermore, hypomethylation was previously associated with cancer ( Nishiyama and Nakanishi, 2021) and other psychiatric disorders, such as schizophrenia ( Shimabukuro et al., 2007) and ADHD in adults ( Müller et al., 2022). Therefore, the overall literature is consistent with our findings pointing to hypomethylation in AUD.

In addition to this evidence, an EWAS pointed out the involvement of the glucocorticoid signaling pathway in AUD, highlighting the importance of the hypothalamic-pituitary-adrenal (HPA) axis in this disorder ( Lohoff et al., 2021). Alcohol stimulates the HPA axis and glucocorticoid receptors, increasing cortisol release. This scenario could be related to the development and severity of AUD ( Blaine and Sinha, 2017). Hence, the inverse relationship between cortisol and GMe ( Müller et al., 2021) could also support the association of AUD and hypomethylation found in our study. On the other hand, other studies suggested hypermethylation levels in individuals with AUD ( Bönsch et al., 2004; Kim et al., 2016), mainly using GMe proxy-based approaches. For instance, Kim *et al.* (2016) assessed the methylation of *Alu* elements, which comprise a third of all methylation sites ( Deininger and Batzer, 1999). Small variations in GMe levels may represent a large number of cytosines differentially methylated ( Breton et al., 2017). Despite the overall evidence indicating a hypomethylation state, the chronic use of alcohol could also lead to a combination of epigenetic and regulatory processes, promoting hypermethylation at specific loci, and contributing to these mixed findings. 

We also investigated the influence of the pattern of alcohol use on DNA methylation levels, and no association was detected. Most of our sample consisted of individuals with heavy alcohol use, and the majority had a daily consumption. Therefore, the uniformly high severity of alcohol consumption within the sample may have precluded the observation of nuances in the methylation pattern. Longitudinal studies showed that the methylation pattern did not change in a short period during detoxification treatment ( Soundararajan et al., 2021). It is possible that the different correlates of GMe status (psychiatric background, HPA axis effects, withdrawal status, and as a direct environmental hazard) could be related to the mixed findings in the relationship between AUD and methylation status. Also, population cohorts may be better suited to assess the impact that the variation in the magnitude of alcohol use, from sporadic to excessive and chronic use, has on GMe levels.

This study had some limitations. First, GMe methylation was assessed from peripheral blood samples, which is considered a limitation in studies evaluating a brain disorder. Although the correlation of blood-brain genome-wide methylation is high (r=0.86) ( Braun et al., 2019), the evaluation of methylation levels directly from the brain remains the desirable choice in psychiatric disorders. On the other hand, alcoholism impacts many organ systems and studies with blood or other tissue samples (e.g., liver) are warranted. Furthermore, DNA methylation can be influenced by several environmental aspects, and unfortunately, not all of them were evaluated in this study, such as stress, diet, and nutritional aspects. Therefore, we cannot rule out the involvement of these factors in GMe levels. Finally, there are some issues regarding our sample that demand a cautious interpretation and the need for independent replications. Our sample included only men, most of them with severe clinical conditions at the beginning of their detoxification treatment. Considering the differences previously reported for sex and AUD prevalence, treatment, and comorbidities patterns ( Agabio et al., 2017; Goh et al., 2022), the homogeneity of our sample can be viewed as a strength. However, we reinforce the need to evaluate women and less severe users in the context of epigenetics and AUD. Individuals with and without AUD differ regarding age and skin color, variables potentially relevant in GMe. However, the association between GMe levels and AUD remained with a significant effect even with adjustment for these variables, reinforcing the need for further studies exploring how these complex variables may interact.

In the present study, a significant effect of AUD was observed in GMe levels. This global hypomethylation observed in individuals with AUD can be considered a biological record of the impact of chronic alcohol use. Identifying connections between epigenetic factors and AUD traits and characteristics is of utmost relevance to unraveling the biological aspects of this disorder. Our findings also represent a possible pathway linking the environmental hazard represented by alcohol use and the multiple organ damages that follow AUD. The complexity of the methylation changes associated with AUD indicates the need for further studies with different clinical profiles, including longitudinal approaches.
